# Butyrate Inhibits Cancerous HCT116 Colon Cell Proliferation but to a Lesser Extent in Noncancerous NCM460 Colon Cells

**DOI:** 10.3390/nu9010025

**Published:** 2017-01-01

**Authors:** Huawei Zeng, David P. Taussig, Wen-Hsing Cheng, LuAnn K. Johnson, Reza Hakkak

**Affiliations:** 1 United States Department of Agriculture, Agricultural Research Service, Grand Forks Human Nutrition Research Center, Grand Forks, ND 58203, USA; dtaussig@gmail.com (D.P.T.); luann.johnson@ars.usda.gov (L.K.J.); 2 Department of Food Science, Nutrition and Health Promotion, Mississippi State University, Starkville, MS 39762, USA; wcheng@fsnhp.msstate.edu; 3 Departments of Dietetics and Nutrition, University of Arkansas for Medical Sciences, Little Rock, AR 72205, USA; RHakkak@uams.edu; 4 Arkansas Children Research Institute, Little Rock, AR 72202, USA

**Keywords:** apoptosis, butyrate, colon cancer, cell proliferation, microbiota

## Abstract

Butyrate, an intestinal microbiota metabolite of dietary fiber, exhibits chemoprevention effects on colon cancer development. However, the mechanistic action of butyrate remains to be determined. We hypothesize that butyrate inhibits cancerous cell proliferation but to a lesser extent in noncancerous cells through regulating apoptosis and cellular-signaling pathways. We tested this hypothesis by exposing cancerous HCT116 or non-cancerous NCM460 colon cells to physiologically relevant doses of butyrate. Cellular responses to butyrate were characterized by Western analysis, fluorescent microscopy, acetylation, and DNA fragmentation analyses. Butyrate inhibited cell proliferation, and led to an induction of apoptosis, genomic DNA fragmentation in HCT116 cells, but to a lesser extent in NCM460 cells. Although butyrate increased H3 histone deacetylation and p21 tumor suppressor expression in both cell types, p21 protein level was greater with intense expression around the nuclei in HCT116 cells when compared with that in NCM460 cells. Furthermore, butyrate treatment increased the phosphorylation of extracellular-regulated kinase 1/2 (p-ERK1/2), a survival signal, in NCM460 cells while it decreased p-ERK1/2 in HCT116 cells. Taken together, the activation of survival signaling in NCM460 cells and apoptotic potential in HCT116 cells may confer the increased sensitivity of cancerous colon cells to butyrate in comparison with noncancerous colon cells.

## 1. Introduction

Colon cancer is the third most frequently occurring cancer in men and women in the United States. In 2016 about 134,490 people are predicted to be diagnosed with colorectal cancer in the US, and it is likely that half the Western population will develop at least one colorectal tumor by the age of 70 years [1,2]. It has been reported that high intake of dietary fiber and resistant starches reduces the risk of colon cancer in human populations and animal models [3,4]. This effect may be related to butyrate, a short chain fatty acid (SCFA), which is produced in the colonic lumen by the bacterial fermentation of dietary fiber [3,4,5,6]. Colonic luminal SCFA concentration can reach 10 mmol/L when humans consume diets containing moderate levels of fiber [7,8]. Conceivably, there is continuous butyrate exposure in the colonic epithelium, and butyrate may exert several anticarcinogenic effects through the modulation of colon cell proliferation and apoptosis [9,10,11,12].

A successful chemoprevention agent should have a minimal effect on normal cells but a strong inhibitory effect on cell proliferation and carcinogenic pathways in cancer cells. While much has been studied on the effect of butyrate on colon cancer cells, little is known about its effect on noncancerous cells, which is essential for understanding butyrate’s anticancer properties. Several studies have shown that histone deacetylase (HDAC) activity represses transcriptional activity by condensing the chromatin package leading to an epigenetically mediated silencing of tumor suppressor genes like p21 [13,14]. Butyrate is an HDAC inhibitor (HDACi) and a potential anti-tumor agent. HDACi strongly activates the expression of the cyclin-dependent kinase inhibitor p21, a tumor suppressor [13,15]. In addition, previous data have shown that the extracellular-regulated kinase 1/2 (ERK1/2) and myelocytomatosis (c-Myc) signaling pathways are both required to drive cell cycle progression during cell proliferation [16,17,18]; c-Myc may also modulate p21 expression [19,20]. To study the effects of butyrate on these signaling pathways related to colon cancer proliferation, two human colon cell lines were employed in this study. The NCM460 colon cell line is an epithelial cell line which is noncancerous and derived from normal colon mucosa [21]. This cell line has not been infected or transfected with any genetic information, and is widely used because there are few other noncancerous colon cell lines [21]. Although there are several cancerous colon cell lines (e.g., HCT116, HT29, and Caco-2) available, most of these cell lines are derived from adenocarcinoma, and only the HCT116 cell line is derived from carcinoma [22,23]. It is known that adenocarcinoma develops in glands whereas carcinoma originates in the epithelial tissue, and there are differences between colorectal carcinoma and advanced adenomas [24]. Given the fact that NCM460 and HCT116 cells were both originally derived from (male adult) colon epithelial tissues and are the same cell subtypes, we believe that NCM460 and HCT116 cells are the best cell-line pair to use in our present study.

In view of the critical role of butyrate in colon cancer prevention [3,4,5,6], we hypothesize that butyrate inhibits cancerous cell proliferation but to a lesser extent in noncancerous colon cells through signaling pathways regulating apoptosis and cellular survival [21,22]. Colon crypt cells divide rapidly and travel to the top of the epithelium where they differentiate, proliferate, and undergo cell cycle progression and apoptosis within 48 h. Thus, we focused on the effects of butyrate on colon cell growth and apoptosis for up to 48 h in the present study whereas signaling molecules were examined at early time points (e.g., 1.5 h) to limit the bystander effect.

## 2. Materials and Methods

### 2.1. Cell Cultures

HCT116 colorectal carcinoma cells were obtained from American Type Culture Collection and maintained in Dulbecco’s Modified Eagle Medium (DMEM) (Invitrogen, Carlsbad, CA, USA) with 10% fetal bovine serum (FBS; Sigma Chemical Co., St. Louis, MO, USA). The nontransformed, noncancerous colon NCM460 cells derived from human normal colon mucosa [21,25] were maintained in M3 Base medium (INCELL Corp., San Antonio, TX, USA) with 10% FBS. Sodium butyrate (purity > 98.5%) was purchased from Sigma Chemical Corporation (St. Louis, MO, USA). Stock cells were passaged twice weekly at ~<80% confluency in Ca-Mg-free Hanks’ balanced salt solution (Sigma Chemical Co., St. Louis, MO, USA) containing 0.25% trypsin (Invitrogen) and 1 mmol/L ethylenediamine tetra-acetic acid (EDTA). Cell viability was determined by trypan blue exclusion based on hemocytometer counts and cells were incubated in a humidified chamber at 36.5 °C with 5% CO_2_. Cultures were tested and found to be mycoplasma free [26]. HCT116 cells at passages 22–40 and NCM460 cells at passages 34–50 were used. Importantly, both HCT116 and NCM460 cell lines were grown in DMEM medium with 10% FBS in all subsequent assays. 

### 2.2. Cell Count/Growth Assay

HCT116 and NCM460 cell lines were both cultured in DMEM medium with 10% FBS, harvested with 0.25% trypsin (Invitrogen) and 1 mmol/L EDTA, and resuspended in 1 mL medium. Cells were then diluted 1:2 (or 1:4) in 0.2% trypan blue and counted in duplicate using a hemocytometer. At least 200 cells per sample were counted. 

### 2.3. Apoptosis Analysis

Apoptosis was analyzed using a Guava Nexin™ Kit (Guava Technologies, Inc. Hayward, CA, USA). HCT116 and NCM460 cell lines were tryspinized, and then suspended in growth media (DMEM with 10% FBS). Annexin V is a calcium-dependent phospholipid binding protein with high affinity to phosphatidylserine (PS), which has translocated from the internal to external side of the cell membrane upon induction of apoptosis. The cell impermeant dye 7-amino-actinomycin D (7-AAD) is included in the assay kit as an indicator of membrane structural integrity [27]. Therefore, [Annexin V (+), 7-AAD (−)] cells are in the early stages of apoptosis, and [Annexin V (+) and 7-AAD (+)] cells are in the late stages of apoptosis. At least 2000 single cell events per sample were analyzed by the Guava PCA System (Hayward, CA, USA).

### 2.4. DNA Fragmentation Assay

Each DNA sample was extracted from about 1,000,000 cells by overnight incubation at 50–55 °C in a lysis buffer (50 mmol/L Tris-HCl, pH 8.0, 10 mmol/L EDTA, 150 mmol/L NaCl, 100 μg/mL proteinase K). These DNA samples were recovered by isopropanol precipitation, resuspended in Tris-EDTA-RNase (6 units/mL), analyzed using 1.9% agarose gels, and visualized by ethidium bromide staining. The intensity signals of genomic DNA fragmentation were analyzed by the UVP Bioimaging Systems (Upland, CA, USA).

### 2.5. Western Blotting Analysis

After butyrate treatment for 1.5 or 15 h, adherent cells were scraped, pooled with the detached cells in 5 mL media, and then these cells were collected by centrifugation at 350× *g* for 10 min at 4 °C. At least four independent experimental cell sample sets were collected. The cell pellet (about 1,000,000 cells) was washed once in ice-cold PBS and lysed in a cell lysis buffer (20 mmol/L Tris-HCT, pH 7.5, 150 mmol/L NaCl, 1 mmol/L Na2EDTA, 1 mmol/L EGTA, 1% Triton, 2.5 mmol/L sodium pyrophosphate, 1 mmol/L Na_3_VO_4_, 1 μg/mL leupeptin, 1 mmol/L phenylmethylsulfonyl fluoride) (Cell Signaling Technology, Inc., Danvers, MA, USA). After 15 s sonication, the cell lysate was centrifuged at 14,000× *g* for 30 min at 4 °C. The supernatant was designated as whole cell protein extract and kept at −80 °C. The protein concentration was quantified by the Bradford dye-binding assay (Bio-Rad laboratories, Richmond, CA, USA). Protein extracts with equal amount (~40 μg) were resolved over 4%–20% Tris-glycine gradient gels under denaturing and reducing conditions and electroblotted onto polyvinylidene difluoride (PVDF) membranes (Invitrogen, Carlsbad, CA, USA). Membrane blots were blocked in phosphate-buffered saline (PBS)—0.05% Tween (*v*/*v*) supplemented with 1% (*wt*/*v*) nonfat dry milk (BioRad, Hercules, CA, USA) overnight at 4 °C. Membranes were probed with antibodies against c-Myc (Epitomics, Inc., Burlingame, CA, USA), phosphorylated ERK1/2 (p-ERK1/2), ERK1/2, acetyl-Histone H3 (Lys9), and p21 antibodies (Cell Signaling Technology, Inc., Danvers, MA, USA), and then incubated with an anti-mouse/rabbit (1:3000 dilution) horseradish peroxidase (HRP)-conjugated secondary antibody (Cell Signaling Technology, Inc., Danvers, MA, USA) in blocking solution for 1 h at room temperature. Blots were washed as above and proteins were incubated with an ECL plus kit (Amersham Pharmacia Biotech, Piscataway, NJ, USA) and imaged by the Molecular Dynamics Image-Quant system (Sunnyvale, CA, USA).

### 2.6. Immunfluorescent Staining

HCT116 and NCM460 colon cells were seeded on microscope slides (about 200,000 cells per cell culture chamber slide) in DMEM media supplemented with 10% FBS under an atmosphere of 5% CO_2_ at 37 °C overnight. For the observation of p21 and its nuclear localization, the cells were pretreated with butyrate for 15 h. After the treatment, cells were fixed using 4% paraformaldehyde for 15 min, they were permeabilized with ice-cold 100% methanol for 10 min at −20 °C with PBS rinse for 5 min. Cells were then blocked with 10% goat serum (Sigma Chemical Corporation, St. Louis, MO, USA) for 1 h, and then incubated with an anti-p21 antibody (Cell Signaling Technology, Inc., Danvers, MA, USA) overnight at 4 °C. Cells, after washing with PBS, were incubated with anti-rabbit Immunoglobulin G (IgG, H and L), F(ab’)_2_ Fragment (Alexa Fluor^®^ 488 Conjugate) (green fluorescence) (Cell Signaling Technology, Inc., Danvers, MA, USA) for 1 h at room temperature with propidium iodide (PI) (25 μg/mL). Finally, fluoroshield with PI (Sigma Chemical Corporation, St. Louis, MO, USA), an aqueous mounting medium, was used for preserving fluorescence and producing a red fluorescence as counter stain for overall cell morphology. The fluorescence images and intensity quantification of at least more 2000 cells (per sample) were analyzed by Nikon E400 microscope and Image Pro Plus version 9.1 (North Central Instruments, Plymouth, MN, USA). 

### 2.7. Statistical Analysis

Results are given as means ± SDs. The concentration of butyrate needed to inhibit cell growth by 50% (IC_50_) was estimated by fitting a three-parameter logistic model to percent inhibition using concentration as the independent variable. The resulting model was used to predict the concentration at which 50% inhibition was expected to occur. Proc NLIN in SAS was used to fit the model. The HCT116 and NCM460 data for 24 h and 48 h were analyzed by two-way analysis of variance with cell type (HCT116 or NCM460), treatment concentration, and their interaction as fixed effects and experiment as a blocking factor. Data were log-transformed prior to analysis in order to test whether changes were proportional across treatment concentrations. Dunnett’s multiple comparison procedure was used to compare individual HCT116 or NCM460 group means with their respective control group (untreated cells). The Proc Mixed procedure in SAS v. 9.4 (SAS Institute, Inc., Cary, NC, USA) was used for all analyses. Differences with a *p* value < 0.05 were considered statistically significant.

## 3. Results

### 3.1. Differential Effects of Butyrate (NaB) on Cell Growth

**Figure 1:**
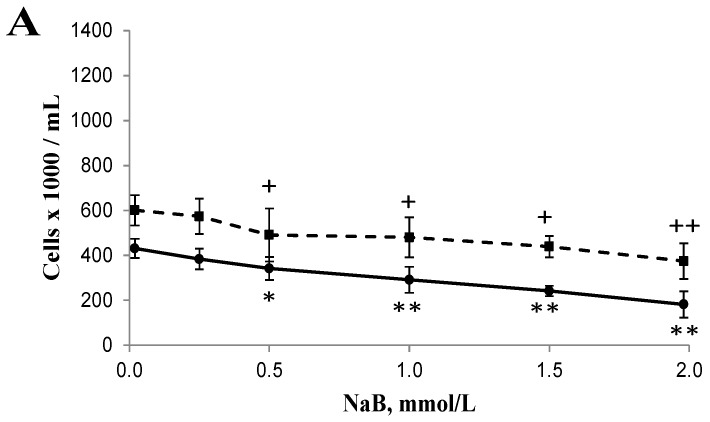

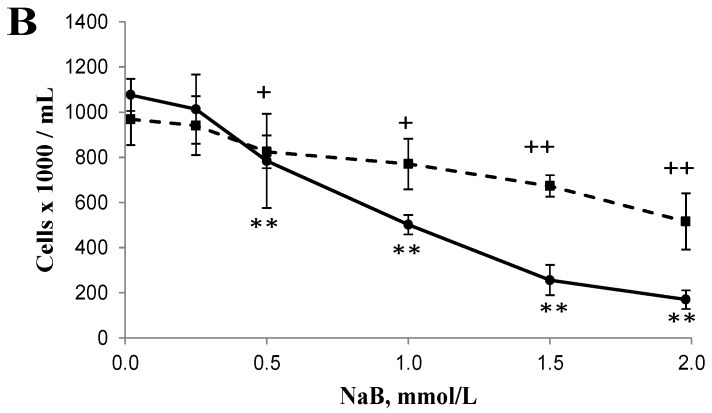
Effect of sodium butyrate (NaB) treatment for (**A**) 24 h and (**B**) 48 h on the growth of cancerous HCT116 (solid lines) and non-cancerous NCM460 (dashed lines) colon cells. Values are means ± SD, *n* = 5 to 6. There was a significant interaction between cell type and concentration at 24 h (*p* = 0.01) and at 48 h (*p* < 0.0001) by two-way ANOVA. * Different from HCT116 control (0 mmol/L NaB); * *p* < 0.05, ** *p* < 0.0001. + Different from NCM460 control (0 mmol/L NaB); ^+^ *p* < 0.05, ^++^ *p* < 0.0001.

The cell growth rate was inhibited in a dose-dependent manner with a maximum of 58% at 24 h, and 84% at 48 h, respectively, in HCT116 cells treated with 0.5, 1, 1.5, or 2 mmol/L NaB when compared with that of untreated cells (Figure 1). In contrast, the cell growth rate was inhibited to a lesser extent in a dose-dependent manner with a maximum of 38% at 24 h, and 47% at 48 h, respectively, in NCM460 cells treated with 0.5, 1, 1.5, or 2 mmol/L NaB when compared with that of untreated cells (Figure 1). At 48 h, the IC50 of butyrate to inhibit HCT116 cell growth was 0.91 mmol/L, and the 95% confidence interval around this estimate was (0.81, 1.02). In contrast, the IC50 of butyrate to inhibit NCM460 cell growth was greater than 2 mmol/L; we could not precisely determine the value because 2 mmol/L was the highest concentration of NaB used in this study (Figure 1B).

### 3.2. Differential Effects of Butyrate (NaB) on Apoptosis

**Figure 2:**
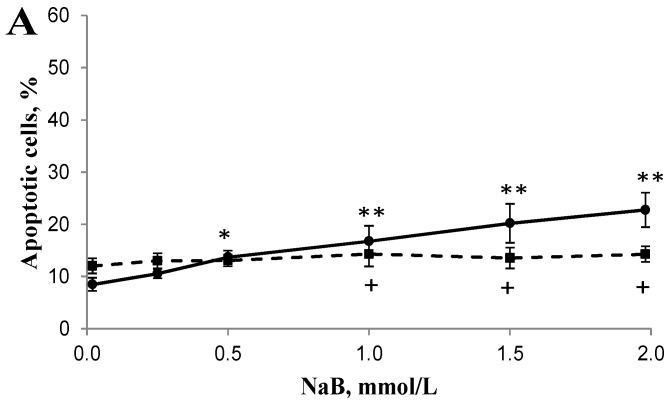

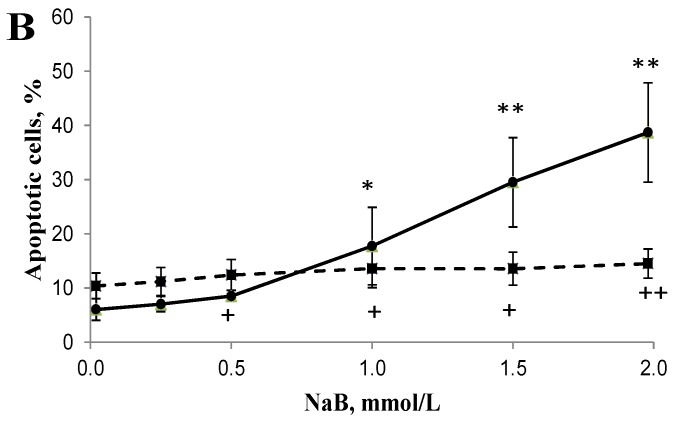
Effect of sodium butyrate (NaB) treatment for (**A**) 24 h and (**B**) 48 h on the apoptosis (including both early and late apoptosis) of cancerous HCT116 (solid lines) and non-cancerous NCM460 (dashed lines) colon cells. Values are means ± SD, *n* = 5 to 6. There was a significant interaction between cell type and concentration at 24 h (*p* < 0.0001) and at 48 h (*p* < 0.0001) by two-way ANOVA. * Different from HCT116 control (0 mmol/L NaB); * *p* < 0.05, ** *p* < 0.0001. + Different from NCM460 control (0 mmol/L NaB); ^+^ *p* < 0.05, ^++^ *p* < 0.0001.

Apoptotic cells (including both early and late apoptosis) were increased in a dose-dependent manner with a maximum 1.7 fold increase at 24 h, and 5.4 fold increase at 48 h, respectively, in HCT116 cells treated with 1, 1.5, or 2 mmol/L NaB when compared with that of untreated cells (Figure 2). In contrast, apoptotic cells were increased in a dose-dependent manner with a maximum 0.2 fold increase at 24 h, and 0.4 fold increase at 48 h, respectively, in NCM460 cells treated with 1, 1.5, or 2 mmol/L NaB when compared with that of untreated cells (Figure 2). Furthermore, the early and late apoptotic cells were also increased in a dose-dependent manner, respectively. The percentage of early apoptotic cells was greater (*p* < 0.05) in HCT116 cells treated with 1, 1.5, or 2 mmol/L NaB when compared with that of untreated cells (9.05 ± 5.07, 16.05 ± 5.76, 21.63 ± 6.84 vs. 2.36 ± 0.75, respectively) at 48 h. The percentage of early apoptotic cells was greater (*p* < 0.05) in NCM460 cells treated with 0.5, 1, 1.5, or 2 mmol/L NaB when compared to untreated cells (5.23 ± 1.45, 6.21 ± 1.86, 6.37 ± 2.04, 6.59 ± 1.83 vs. 3.91 ± 1.27) at 48 h. Similarly, the percentage of late apoptotic cells was greater (*p* < 0.05) in HCT116 cells treated with 1, 1.5, or 2 mmol/L NaB when compared with that of untreated cells (8.89 ± 2.76, 13.48 ± 2.78, 17.09 ± 3.09 vs. 3.70 ± 1.36, respectively) at 48 h. In contrast, in NCM460 cells, the percentage of late apoptotic cells was greater (*p* < 0.05) only in cells treated with the highest concentration of NaB (2 mmol/L) when compared to untreated cells (7.93 ± 1.02 vs. 6.49 ± 1.20) at 48 h.

### 3.3. Differential Effects of Butyrate (NaB) on DNA Fragmentation

**Figure 3:**
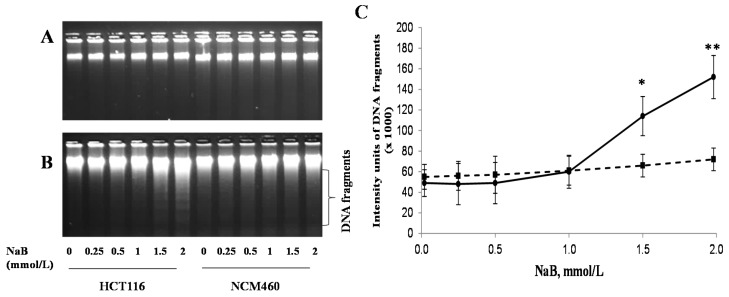
Effect of sodium butyrate (NaB) on the genomic DNA fragmentation of cancerous HCT116 and non-cancerous NCM460 colon cells. A representative DNA image showing DNA fragmentation at (**A**) 1.5 h; (**B**) 15 h; (**C**) the intensity signals of genomic DNA fragmentation (at 15 h) of cancerous HCT116 (solid lines) and non-cancerous NCM460 (dashed lines) colon cells were analyzed by the UVP Bioimaging Systems (there was no DNA fragmentation at 1.5 h). Values are means ± SD, *n* = 4. There was a significant interaction between cell type and concentration (*p* < 0.0001) by two-way ANOVA. * Different from HCT116 control (0 mmol/L NaB); * *p* < 0.05, ** *p* < 0.0001.

The intensity of genomic DNA fragmentation was increased by 1.3 and 2.1 fold in HCT116 cells treated with 1.5 or 2 mmol/L NaB, respectively, for 15 h (Figure 3B) but not 1.5 h (Figure 3A) when compared with that of untreated cells (Figure 3). There was no NaB-induced genomic DNA fragmentation at 1.5 or 15 h in NCM460 cells.

### 3.4. Differential Effects of Butyrate (NaB) on Signaling Molecules

**Figure 4:**
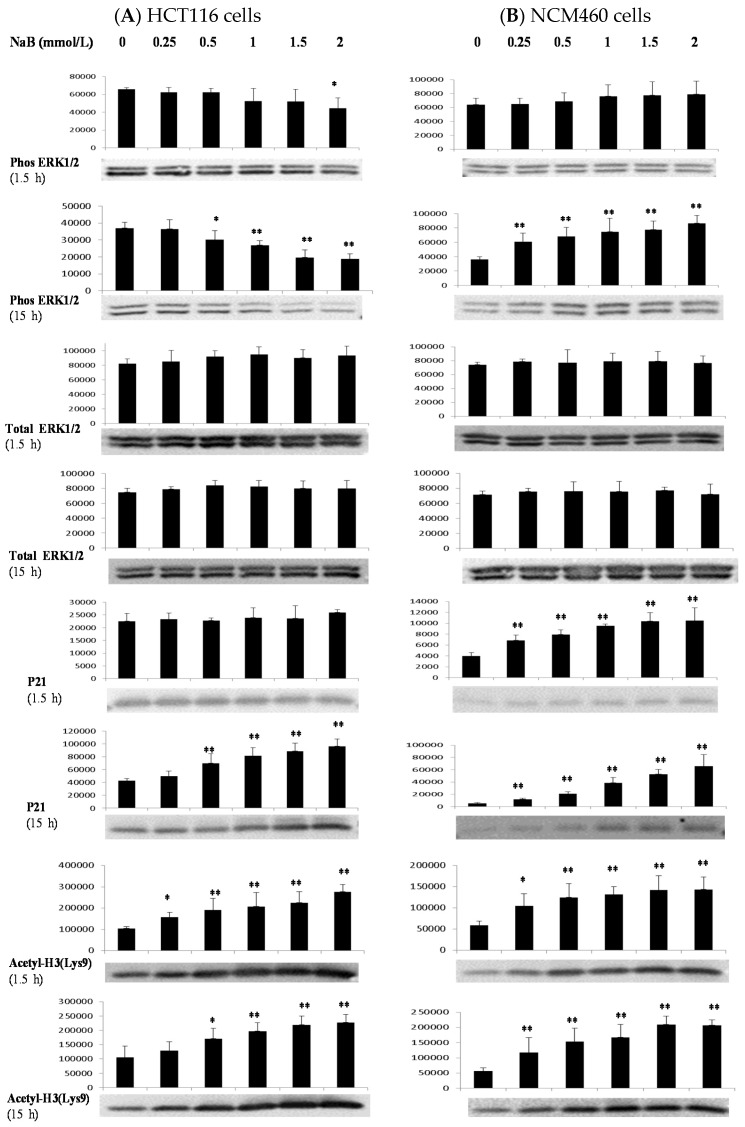

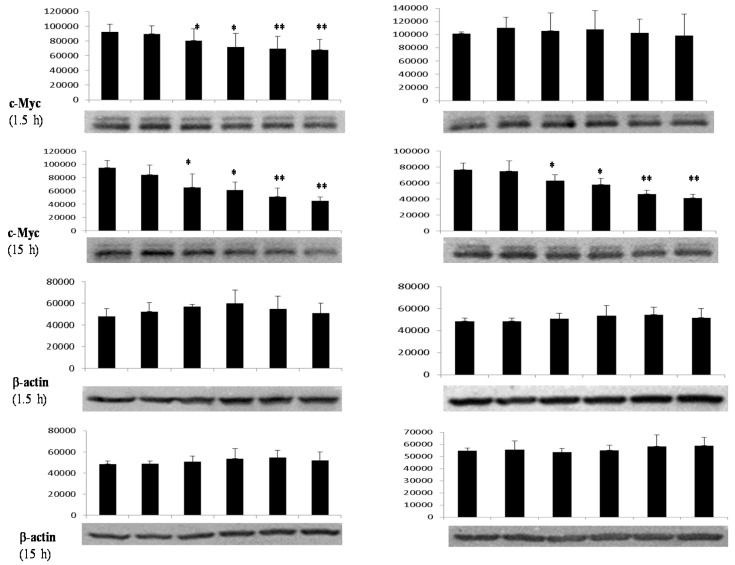
Effect of butyrate on the cell-proliferation proteins. Western blot analyses of the effects of sodium butyrate (NaB) treatment for 1.5 h or 15h on intracellular signaling proteins (densitometric units) in (**A**) HCT116 colon cells and (**B**) NCM460 colon cells. Values are means ± SD, *n* = 4, * Different from control (0 mmol/L NaB); * *p* < 0.05, ** *p* < 0.0001. A representative Western blotting image was from four independent experiments for a given antibody assay.

While the p-ERK1/2 level was decreased dose-dependently at 15 h after treatment with NaB in HCT116 cells, the opposite trend was observed in NCM460 cells (Figure 4). Except for the p21 level at 1.5 h in HCT116 cells, there were NaB dose-dependent increases in levels of p21 and acetyl-H3 at Lys 9 at 1.5 and 15 h in both HCT116 and NCM460 cells (Figure 4). Except for the level of acetyl-H3 at Lys 9 in HCT116 cells, the extent of p21 and acetyl-H3 at Lys 9 induction was greater at 15 h than 1.5 h in both types of cells. In contrast, there were NaB dose-dependent decreases in c-Myc protein level at 1.5 and 15 h in HCT116 cells and 15 h in NCM460 cells (Figure 4). The β-actin and total ERK1/2 protein levels did not differ at 1.5 and 15 h in HCT116 and NCM460 cells because of NaB treatment (Figure 4).

### 3.5. Differential Effects of Butyrate (NaB) on p21 Protein Level and Cellular Localization

**Figure 5:**
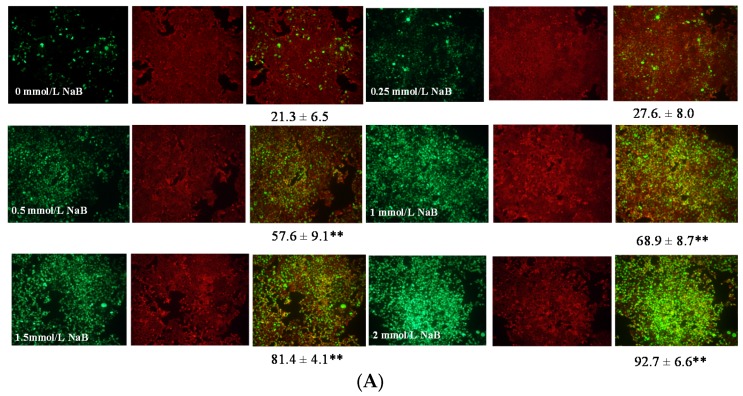

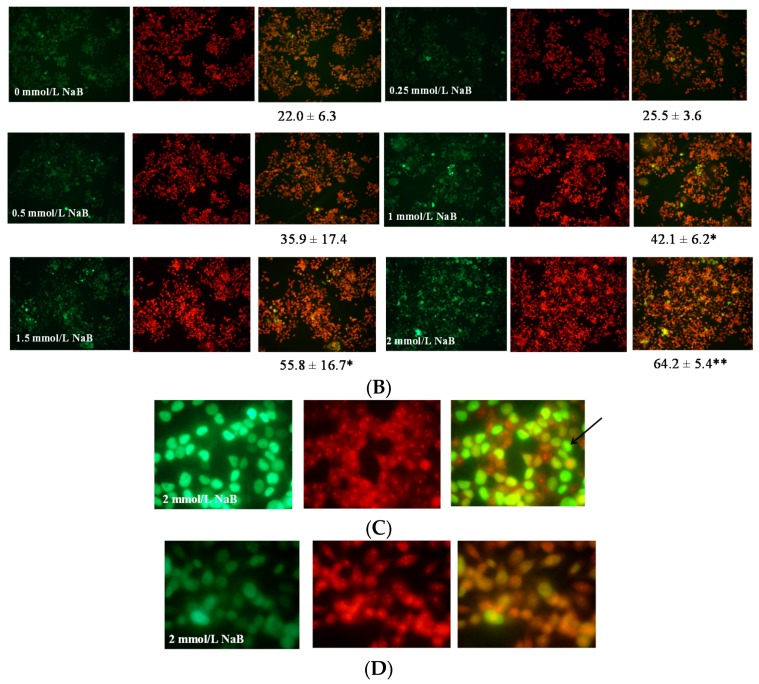
Effect of sodium butyrate (NaB) treatment for 15 h on p21 protein level and distribution at cellular level in HCT116 and NCM460 colon cells. Each sample consists three images: image 1, cells were labeled with anti-p21 antibody, and followed by anti-Rabbit IgG (H and L), F(ab’)_2_ Fragment (Alexa Fluor^®^ 488 Conjugate) (green signals); image 2, cells were mounted by fluoroshield with PI as counter staining for overall cell morphology-background (red signals); image 3, the p21 protein image was superimposed on the respective overall cell morphology-background image to generate the composite image (orange signals). (**A**) HCT cells at 200× magnification; (**B**) NCM460 cells at 200× magnification; (**C**) HCT116 cells with intense p21 expression around the nucleus (arrow) at 1000× magnification; (**D**) NCM460 cells at 1000× magnification. For panel (**A**,**B**) composite images, the area of p21 protein level (green signals) in percentage when compared with that of overall cell morphology-background (red signals). Values are means ± SD, *n* = 4. There was a significant interaction between cell type and concentration (*p* < 0.001) by two-way ANOVA. * Different from control (0 mmol/L NaB); * *p* < 0.05, ** *p* < 0.0001.

The ratio percentage (composite image) of p21 protein level (green signals) vs. overall cell background (red signals) at 15 h was 69%, 81%, and 93%, respectively, in HCT116 cells treated with 1, 1.5, or 2 mmol/L NaB when compared with that of untreated cells. To a lesser extent, the ratio percentage (composite image) of p21 protein level (green signals) vs. overall cell background (red signals ) at 15 h was 42%, 56%, and 62%, respectively, in NCM460 cells treated with 1, 1.5, or 2 mmol/L NaB when compared with that of untreated cells (Figure 5A,B). In addition, the overall p21 protein level (green signals) was greater with intense expression around the nuclei in HCT116 cells when compared with that in NCM460 cells (Figure 5C,D).

## 4. Discussion

Butyrate has been shown to abrogate the S-phase cell cycle checkpoint, and exhibits colon cancer preventive effects through cell proliferation regulation [5,6,28,29,30]. As there are few reports concerning the apoptotic potential, we propose that butyrate plays differential roles in the cell proliferation of noncancerous NCM460 cell and cancerous HCT116 colon cells through cellular signaling modulation. Thus, examining molecular effects of butyrate on cell proliferation/apoptosis in cancerous and noncancerous colon cells is expected to shed light on butyrate’s anticancer mechanism.

It has been reported that butyrate at mmol/L levels in the colon is well within physiological concentrations [7,8]. Our data showed that butyrate (0.25 to 2 mmol/L) was much more effective on inhibiting cell proliferation in cancerous (HCT116) colon cells than in noncancerous (NCM460) colon cells (Figure 1). Similarly, we found, for the first time, that butyrate was effective in inducing apoptosis/DNA fragmentation in HCT116 cells but not NCM460 cells (Figure 2 and Figure 3). This observation suggests that the stronger DNA fragmentation and apoptotic potential may confer the increased sensitivity of cancerous colon cells to butyrate in terms of cell proliferation when compared with that of noncancerous colon cells. In our present apoptosis assay, 7-AAD was excluded from live, healthy cells and early apoptotic cells, but permeated late apoptotic or necrotic cells [27]. It has been demonstrated that secondary necrosis is a natural outcome of the complete apoptotic program (e.g., late apoptotic cells) [31]. Although lactate dehydrogenase (LDH), a cytoplasmic enzyme, is widely used to detect necrosis and secondary necrosis based on plasma membrane leakage, the measurement of 7-AAD positive cells is a powerful approach to directly detect leaky membrane cells [31,32]. As our results showed that the late apoptotic cell population (including necrosis and possible secondary necrosis) was greatly increased in HCT116 cells but to a lesser extent in NCM460 cells due to butyrate treatment, it is conceivable that necrotic cell death may evoke inflammatory responses [32,33]. In the future, clinical samples from patients are needed to examine the impact of inflammatory responses on host pathogenesis in the context of high or low exposure to butyrate. 

The other important aspect is that the cellular signaling molecules underlying the differential effect of butyrate remain to be determined. The ERK1/2 pathway plays a pivotal role in cell proliferation as it is an important cellular signaling component that translates various extracellular signals into intracellular responses through phosphorylation cascades [18,34,35,36]. Moreover, cell-cycle arrest by PD184352 or U0126 requires inhibition of ERK1/2 activation [37]. Thus, the ERK1/2 pathway is recognized as a pro-survival signal and often activated by growth factors [18,34,35]. In this study, butyrate up-regulated ERK1/2 phosphorylation in NCM460 cells (Figure 4). In contrast, butyrate inhibited ERK1/2 phosphorylation in HCT116 cells, which is consistent with the previous report [38] (Figure 4). The opposing effect of butyrate on cell survival signals provides an important mechanistic insight into the observed differential efficacy of cell proliferation and apoptosis in cancerous and noncancerous colon cells.

Appropriate control over cell cycle and proliferation depends on many factors. Cyclin-dependent kinase (CDK) inhibitor p21 (also known as p21 (WAF1/Cip1)) is one of these factors that promotes both cell cycle arrest and proliferation in response to a variety of stimuli [39,40,41]. To provide further mechanistic insights, we examined p21 protein expression, which is known to be the most critical effector of butyrate-induced growth arrest in colon cancer cells [39,42] (Figure 4). Butyrate is known to induce general histone acetylation, specifically, hyperacetylation of the H3 and other species through inhibition of the histone deacetylase enzyme [42,43]. Previous studies have shown that histone hyperacetylation is at least partly responsible for the induction of p21 [42,44]. In addition, the nuclear protein c-Myc, a central regulator of cellular proliferation, activates a multitude of pathways to repress p21 at the transcriptional and post-transcriptional levels [19,20]. We found that butyrate increased histone H3 acetylation at 1.5 and 15 h while it decreased c-Myc expression only at 15 h in NCM460 cells (Figure 4). However, p21 expression was increased at 1.5 and 15 h in NCM460 cells. Therefore, our results suggest that butyrate-related histone acetylation (compared with c-Myc) plays a major role in p21 expression because we could only detect the increase of histone H3 acetylation and p21 but not c-Myc expression in NCM460 cells in early molecular events at 1.5 h. 

The other important aspect of p21 function is that, depending on intracellular localization, p21 is involved in different signaling cascades [39,40,41]. It is generally believed that nuclear p21 is a negative regulator of cell proliferation and a tumor suppressor while cytoplasmic p21 facilitates cell proliferation and inhibits apoptosis [39,40,41]. In addition to high p21 levels in HCT116 cells (Figure 5A,B), our cellular immunofluorescent staining data demonstrated that butyrate-induced p21 protein was located in or surrounding the nuclei of HCT116 cells to a greater extent when compared with that of NCM460 cells (Figure 5C,D). This observation indicates that butyrate induces p21 expression, and may exacerbate the negative role of p21 in HCT116 cell proliferation when compared with that of NCM460 cells. Therefore, butyrate treatment leads to the significant induction of apoptosis and inhibition of cell proliferation in cancerous HCT116 colon cells, but to a lesser extent in the noncancerous NCM460 colon cells. To extrapolate these mechanistic data to all colon cancer cell types and disease stages, we are planning to test other pairs of noncancerous and cancerous colon cell lines. However, there are very limited noncancerous colon cell lines available, which is a challenge. The other approach would be to evaluate clinical samples from patients with possible high or low exposure to butyrate. 

Colon carcinogenesis consists of initiation, promotion, and progression phases [45,46]. Suppression of apoptosis and promotion of colonocyte proliferation are key cellular carcinogenic events as a consequence of dysregulation of molecular signal cascades [46]. Taken together, our findings on the differential roles of butyrate in cell proliferation and the activation of ERK1/2, histone hyperacetylation, and c-Myc, p21 protein abundance and intracellular location in cancerous HCT116 and noncancerous NCM460 colon cells may, at least in part, account for the selective potential of butyrate’s anticancer colon cancer action.
